# Multicohort assessment of plasma metabolic signatures of tuberculosis disease in children

**DOI:** 10.21203/rs.3.rs-7086994/v1

**Published:** 2025-07-29

**Authors:** Mary M. Nellis, Juaneta Luiz, Devan Jaganath, Zaynab Mousavian, Esin Nkereuwem, Peter Wambi, Roger Calderon, Mandar Paradkar, Robert Castro, Rutuja Nerurkar, Molly F. Franke, Aarti Kinikar, Eric Wobudeya, Heather J Zar, Mark Segal, George Sigal, Danielle L. Swaney, Adithya Cattamanchi, Joel D. Ernst, Thomas R. Ziegler, Beate Kampmann, Jeffrey M. Collins

**Affiliations:** Emory University School of Medicine; University of Cape Town; University of California San Francisco School of Medicine; Emory University Rollins School of Public Health; MRC Unit the Gambia; Uganda Tuberculosis Implementation Research Consortium; Advance Research and Health; Byramjee Jeejeebhoy Government Medical College-Johns Hopkins University Clinical Research Site; University of California San Francisco School of Medicine; University of California San Francisco School of Medicine; Harvard Medical School; Byramjee Jeejeebhoy Government Medical College and Sassoon General Hospitals; Uganda Tuberculosis Implementation Research Consortium; University of Cape Town; University of California San Francisco School of Medicine; Meso Scale Diagnostics, LLC; J. David Gladstone Institutes; University of California Irvine; University of California San Francisco School of Medicine; Emory University School of Medicine; MRC Unit the Gambia; Emory University School of Medicine

**Keywords:** Tuberculosis, Diagnostics, Biomarkers, Metabolomics, Children

## Abstract

Current microbiological tests for tuberculosis (TB) disease in children have suboptimal accuracy and rely on respiratory samples which may be challenging to obtain. We sought to use high-resolution metabolomics (HRM) to identify blood-based biomarkers associated with TB disease in children. We analyzed plasma samples from 438 children 0–14 years being evaluated for TB disease in India, Peru, Uganda, The Gambia, and South Africa. All children underwent a standard clinical evaluation and were followed up after 3 months. Children were classified as Confirmed (n = 104), Unconfirmed (n = 108), or Unlikely TB (n = 226) as per NIH consensus definitions. We used liquid chromatography/mass spectrometry for HRM analysis of plasma samples. Differentially regulated metabolic pathways in children with confirmed versus unlikely TB in at least three of the five countries included purine, linoleate, arginine and proline, aspartate and asparagine, and tryptophan metabolism. Controlling for age and study site, we found creatine, alanine, retinol, citrulline, fumarate, and tryptophan to be significantly decreased in children with Confirmed TB disease versus those with Unlikely TB, while cortisol, nicotinamide, and butyrylcarnitine were increased (FDR-corrected p-value < 0.2). Using logistic regression, we found this nine-metabolite signature had an area under the receiver operator characteristic curve (AUC) of 0.72 in the test set of participants with Confirmed and Unlikely TB and an AUC of 0.49 in the Unconfirmed TB group. Of the five cohorts examined, the model performed best among Indian children with Confirmed TB (AUC = 0.84). These results show a nine-metabolite plasma signature has moderate accuracy for identification of Confirmed TB disease in children and could potentially be combined with other non-sputum biomarkers to inform future TB diagnostics.

## Introduction

It is estimated that 1.25 million children develop tuberculosis (TB) disease each year^1^, resulting in more than 200,000 deaths^2^. Only about half of such pediatric TB cases are diagnosed, which contributes to higher TB transmission and mortality^3^. Young children with TB often present with paucibacillary disease and non-specific symptoms, which are barriers to timely diagnosis. Further, many TB diagnostics rely on expectoration of sputum specimens for microbiologic testing, which is particularly challenging in young children, although sputum induction can be used^4^. While the rapid molecular tests Xpert MTB/RIF^5^ and Xpert MTB/RIF Ultra^6^ represent major advances in TB diagnostics, they still require a laboratory and electricity, which are often unavailable at primary care clinics in resource-limited settings where most children initially present with TB symptoms. For these reasons, the World Health Organization (WHO) ranks a rapid, non-sputum, biomarker-based test as the highest priority need for new TB diagnostics^7^. To make progress toward the goal of developing a rapid, non-sputum, point-of-care test for TB disease, it is necessary to identify new biomarkers from more easily obtained clinical samples such as blood^8^.

High-resolution metabolomics (HRM) is an emerging platform for biomarker discovery in TB disease^9^. Innovations in liquid chromatography-mass spectrometry (LC/MS) paired with advanced computational pipelines now allow for simultaneous measurement of thousands of small molecule metabolites in biofluids^10,11^. In adults with TB disease, this has led to several new plasma biomarker candidates, most notably, tryptophan, kynurenine, and retinol^12–15^. While there is some evidence that these metabolic signatures may also have diagnostic potential in children^16^, studies to date have been small with limited geographic diversity and compared children with TB to asymptomatic controls^17^. Further, these studies have been underpowered to evaluate differential metabolic responses across age groups and geographic locations. This is particularly relevant for young children, who may have distinct host response pathways relative to older children and/or adults^18^. Thus, it is essential to evaluate the diagnostic potential of plasma metabolic signatures in children with TB disease in different age groups and across diverse geographic areas. Furthermore, plasma metabolic signatures can provide insight into mechanisms of host responses and may inform development of host-directed therapies.

To evaluate metabolomics for TB biomarker discovery in children, we performed untargeted HRM on previously collected plasma samples from children presenting with presumptive TB disease. Using NIH consensus clinical diagnostic criteria, we compared plasma metabolic signatures in participants with Confirmed and Unconfirmed TB disease versus those with Unlikely TB. Our goal was to discover metabolic TB biomarkers that could be used to detect TB disease across diverse pediatric populations.

## Methods

We analyzed archived plasma samples collected from children less than 15 years old evaluated for pulmonary TB disease and enrolled in The Gambia, Peru, South Africa, India, and Uganda. Children were included if they had either a confirmed pulmonary TB diagnosis (positive Xpert MTB/RIF, Xpert Ultra or culture) or a clinical suspicion of intra-thoracic TB on the basis of having unexplained cough of any duration and one or more of the following symptoms/signs: 1) Weight loss or poor weight gain/failure to thrive, 2) Unexplained fever > 1 week; 3) Unexplained lethargy or reduced playfulness > 1 week; 4) Abnormal chest x-ray; 5) positive tuberculin skin test (TST), or 6) Contact with an individual with TB disease. Children already receiving anti-TB treatment for more than 72 hours, treated for TB disease within the last year, taking anti-TB prophylaxis or unable to provide specimens for mycobacterial culture were excluded from the study. All children completed a standard TB evaluation, including clinical examination, chest x-ray, and respiratory sample collection for Xpert MTB/RIF molecular testing and mycobacterial liquid culture. All children had follow-up after 2–3 months and were assessed for clinical response to any treatment. Improvement at the follow-up visit was defined as weight gain and improvement or resolution of symptoms or signs, compared to the enrollment measurements. Participants were then classified according to NIH consensus definitions as Confirmed, Unconfirmed, or Unlikely TB. Confirmed TB was defined as having a positive Xpert MTB/RIF Ultra or positive culture for *M. tuberculosis*. Unconfirmed TB cases did not have microbiological confirmation of TB but had signs and symptoms of TB disease with other clinical signs or risk factors suggestive of TB and improved with TB treatment. Children categorized as having Unlikely TB did not have microbiological evidence of TB disease and were not clinically diagnosed with TB.

HIV testing was done in all children unless the HIV status of the child was already known; all children living with HIV (CLHIV) also had a CD4 count performed. World Health Organization 2007 growth standards were used to calculate age standardized z scores, and stunting was classified as height-for-age z-score of less than − 2, while underweight was classified as weight-for-age z-score of less than − 2. Blood was collected in either ethylenediaminetetraacetic acid (Peru and Uganda), lithium heparin (The Gambia and India), or sodium heparin (South Africa) -containing tubes, immediately placed on ice, and transferred to an on-site laboratory, where plasma was separated by centrifugation and frozen at − 80 degrees C. Plasma collected in a single type of anticoagulant was not available across sites. For this analysis, we selected a random sample of plasma specimens in a ratio of 1:1:2 of Confirmed TB, Unconfirmed TB, and Unlikely TB, as defined above.

### Human subjects approval

All caregivers completed a written informed consent, including for storage of samples for future studies, and children above seven years of age completed an assent. The studies were approved by the Mulago Hospital Ethics Research Committee, Gambian Government and MRC joint ethics committee, London School of Hygiene and Tropical Medicine, Institutional Ethics Committee for Research of National Institute of Health - Peru, Human Research Ethics committee of the Faculty of Health Sciences, University of Cape Town, Ethics Committee at BJ Government Medical College, Johns Hopkins University (JHU) Institutional Review Board, and the University of California, San Francisco (UCSF) IRB. All study procedures and experiments were performed in accordance with relevant guidelines and regulations.

### Metabolomics analysis

After separating deidentified plasma samples based on the anticoagulant used for collection, an equal distribution of samples from participants with Confirmed TB, Unconfirmed TB, and Unlikely TB were randomly allocated to into blocks of 40 prior to transfer to the analytical laboratory where personnel were blinded to clinical and demographic data^12^. We performed high-resolution plasma metabolomics using a standardized method, which has been published previously.^12,15,19^ Briefly, 50 μL of thawed plasma was treated with 100 μl acetonitrile (2:1, v/v) and an internal isotopic standard mixture (3.5 μL/sample) consisting of 14 stable isotopic chemicals for quality control.^20^ Samples were mixed, placed on ice for 30 minutes, and centrifuged. The supernatant was then analyzed in triplicate using an Orbitrap Q Exactive Mass Spectrometer (Thermo Scientific, San Jose, CA, USA) with dual Hydrophilic Interaction Liquid Chromatography (HILIC) positive and C18 negative liquid chromatography (Higgins Analytical, Targa, Mountain View, CA, USA, 2.1 × 10 cm) with a formic acid/acetonitrile gradient. The mass spectrometer was operated over a scan range of 85 to 1275 mass/charge (*m/z*) and data was stored as.Raw files.^21^ Data were extracted and aligned using apLCMS^22^ and xMSanalyzer^23^ with each feature defined by specific *m/z* value, retention time and integrated ion intensity.^21^ Identities of targeted metabolites were confirmed and quantified by accurate mass, MS/MS and retention time relative to authentic standards.^24^

### Statistics

Statistical comparisons of metabolite intensity values (abundance) and metabolite concentrations were performed in R version 4.2.2. For site-specific pathway analyses, metabolite intensity values were log_2_ transformed and compared between groups using linear regression, controlling for age (< 5 and ≥ 5 years).^25^ Metabolic pathway enrichment analysis was performed using *mummichog*, a Python-based informatics tool that leverages the organization of metabolic networks to predict functional changes in metabolic pathway activity.^12,26,27^ For the final biomarker analysis, metabolite concentrations were log_2_ transformed and compared between groups using linear regression, controlling for age (< 5 and ≥ 5 years) and study site.^25^ A Benjamini-Hochberg false discovery rate (FDR) procedure-corrected p-value (q-value) of less than or equal to 0.2 was used to select metabolites for inclusion in the logistic regression model^28^. To evaluate the model prediction accuracy, children with Confirmed TB and Unlikely TB were randomly divided into a training (70%) and test (30%) data set, respectively. All prediction models were assessed by calculating the area under the receiver operator characteristic curve (AUC). For correlation analyses, plasma metabolite concentrations were normalized using log transformation. For the machine learning analysis, we used the removeBatchEffect function from the Limma R package to eliminate site-specific effects in the data^25^. Feature selection was performed using the randomForestSRC R package^29^ with classification accuracy assessed using both random forest and support vector machine (SVM) models implemented in the caret R package^30^.

## Results

We performed HRM on plasma samples from 438 children being evaluated for TB disease in 5 countries: Peru, Uganda, India, The Gambia, and South Africa (Table 1). After clinical evaluation and follow up, 104 (24%) were classified as having Confirmed TB disease, 108 (25%) as Unconfirmed TB disease, and 226 (51%) as Unlikely TB disease. Two hundred and two (46%) study participants were female, which was similar at all study locations. There were 28 CLHIV (6%), nearly all of whom were enrolled at the South Africa and Uganda study sites.

### Metabolic Pathway Analysis

Metabolomics was performed on archived plasma samples from each country. Plasma samples had been collected in different anticoagulants and were therefore organized into batches according to the anticoagulant used to minimize analytic variability (see Methods for full details). Using untargeted HRM, we detected > 9800 features in positive ionization mode using HILIC and > 7600 features in negative ionization mode using C18 chromatography. Only features present in at least 50% of the samples in each batch were included in downstream analyses. We first compared the metabolome of children with Confirmed TB disease versus those with Unlikely TB controlling for age and sex. For those samples collected using the same anticoagulant and analyzed together (India and The Gambia; Peru and Uganda), we found a greater number of differentially abundant metabolic features when study sites were analyzed separately (Figure S1). The smaller number of significant metabolites despite the larger sample sizes when combining countries suggested strong population-specific metabolic responses to TB disease. After stratifying the analysis by country, we found 1490 features in Peru, 546 features in Uganda, 1493 features in India, 2353 features in The Gambia, and 583 features in South Africa that were differentially abundant between children with Confirmed and Unlikely TB (p < 0.05).

**Figure 1:**
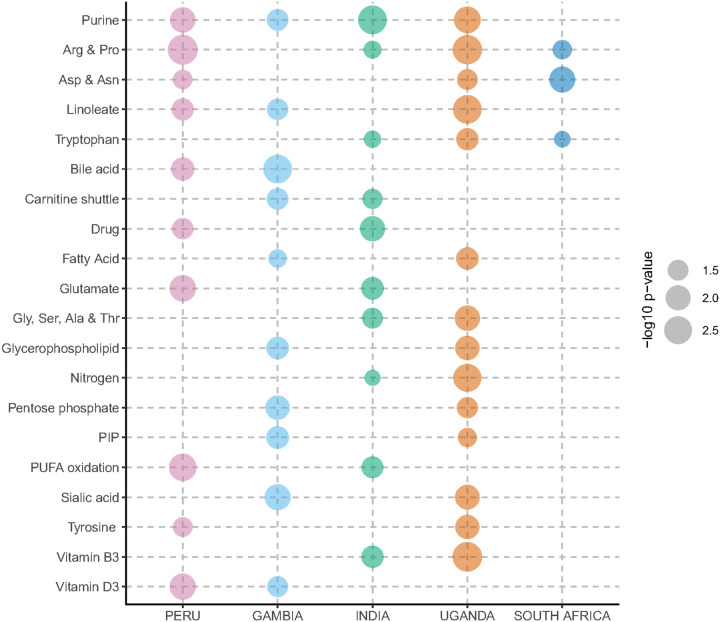
Metabolic pathway changes in children with Confirmed versus Unlikely Tuberculosis – Bubble plot showing metabolic pathways significantly different between children with Confirmed TB disease versus those with Unlikely TB disease in at least two of the following countries: Peru (pink), The Gambia (light blue), India (green), Uganda (orange), and South Africa (dark blue). Each metabolic pathway is shown on the y-axis and each country on the x-axis. The size of each dot represents the -log10 p-value of each pathway.

We then performed a country-level metabolic pathway analysis on all metabolites significantly differentiating children with Confirmed TB disease from those with Unlikely TB. While distinct metabolic pathways distinguished children with Confirmed TB in different countries, several significantly regulated pathways were common among multiple countries (Fig. 1). Pathways involving metabolism of purine, arginine and proline were significantly different in children with Confirmed TB from at least four of the five countries analyzed. Additional pathways that differed significantly among children with Confirmed versus Unlikely TB in at least three countries included linoleate, aspartate and asparagine, and tryptophan metabolism.

### Targeted Metabolite Analysis

**Figure 2:**
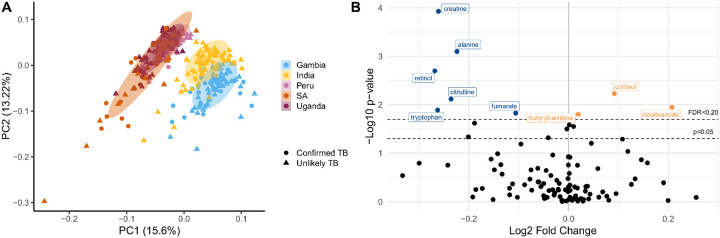
Differentially abundant metabolites in children with Confirmed versus Unlikely Tuberculosis – (A) Principal component analysis showing the clustering of each country using the targeted analysis of 105 metabolites detected and quantified in all cohorts. (B) Volcano plot showing those metabolites that significantly differed between children with confirmed TB versus those with unlikely TB controlling for age group (<5 years of age, ≥ 5 years of age) and study site using linear regression. The y-axis indicates the -log10 p-value for each metabolite and the x-axis displays the log2 fold change between groups. A false-discovery rate (FDR)-corrected p-value of less than 0.20 was considered statistically significant as indicated by the second dashed black line. Metabolites that were significantly decreased in children with confirmed TB are labelled in dark blue while those that were significantly increased are labelled in orange.

Given the most common metabolic pathways differentiating children with TB disease involved metabolism of lipids and amino acids, we performed a targeted analysis of 105 metabolites that could be quantified among children from all countries, which included all amino acids. Each metabolite was identified and quantified using MS/MS relative to authentic reference standards^24^. Following targeted metabolite quantification, we performed a principal component analysis among participants with Confirmed and Unlikely TB to examine whether there remained strong site-specific differences. While children from Uganda, Peru, and South Africa clustered together, participants from India and The Gambia clustered separately from the other groups as well as each other (Fig. 2A). This suggested that strong site-specific effects in plasma metabolite concentrations remained either due to differences in sample collection tubes or environmental and/or dietary exposures. To determine the metabolites that most differentiated children with Confirmed TB from those with Unlikely TB, we performed a differential expression analysis among children from all study countries controlling for age and study site. We found plasma concentrations of creatine, alanine, retinol, citrulline, tryptophan, and fumarate were significantly decreased in children with Confirmed TB (FDR < 0.2) while cortisol, butyrylcarnitine, and nicotinamide were significantly increased (Fig. 2B).

**Figure 3:**
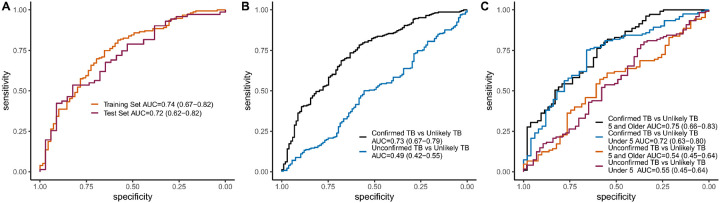
Classification accuracy of a 9-metabolite signature for children with Confirmed and Unconfirmed Tuberculosis – (A) Area under the receiver operator characteristic curve (AUC) for the 9-metabolite signature identified by the differential expression analysis in the training and test data sets of children with Confirmed TB versus those with Unlikely TB. (B) AUC of 9-metabolite signature in the full cohort of children with Confirmed TB as well as those with Unconfirmed TB versus children with unlikely TB. (C) Classification accuracy of the 9-metabolite signature by age group in children with Confirmed or Unconfirmed TB disease versus those with Unlikely TB.

We then created a metabolic signature using logistic regression that included all nine significant metabolites from the differential expression analysis as well as a term for study site to account for site-specific differences. Participants with Confirmed TB and Unlikely TB from all sites were then randomly divided into training (70%) and test (30%) sets to assess the predictive accuracy of a combined metabolic signature. This signature produced reasonable classification accuracy for Confirmed TB versus Unlikely TB in the training set (AUC = 0.74) as well as the test set (AUC = 0.72; Fig. 3A). Classification accuracy dropped off considerably for children with Unconfirmed TB versus Unlikely TB (AUC = 0.49; Fig. 3B). However, this performance was not unexpected given the Unconfirmed TB group did not have any positive microbiologic results for TB and therefore likely represents a mix of children with TB and other respiratory illnesses. Classification accuracy was similar in children under five years versus those five and older with Confirmed versus Unlikely TB (AUC = 0.72 and 0.75 respectively; Fig. 3C). Accuracy was also similar for Unconfirmed versus Unlikely TB (AUC = 0.55 in under five and 0.54 in five and older).

We also tested a metabolite signature that included the kynurenine/tryptophan ratio and retinol, which was shown to have greater accuracy in adults with pulmonary TB^15^. We found this more parsimonious signature performed worse than the 9-metabolite model in children with Confirmed versus Unlikely TB (AUC = 0.59; Figure S2). Model accuracy did improve slightly if only children over 10 were considered (AUC = 0.66), who may have physiology more similar to adults.

### Model performance by country

**Figure 4:**
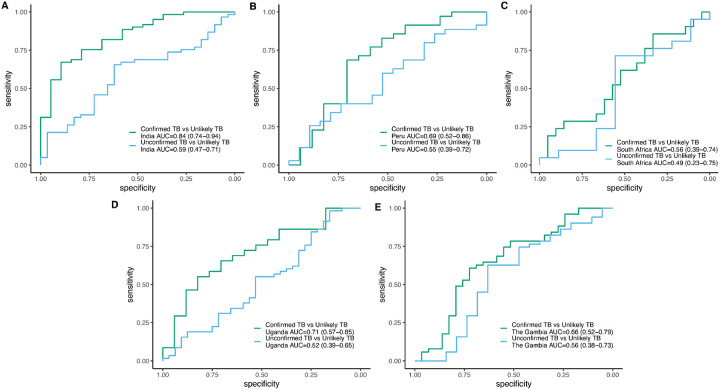
Country-specific classification accuracy of the 9-metabolite signature – Receiver operator characteristic curves demonstrating the classification accuracy of the 9-metabolite signature in children with Confirmed and Unconfirmed TB disease versus those with Unlikely TB in (A) India, (B) Peru, (C) South Africa, (D) Uganda, and (E) The Gambia.

Model performance in individual countries varied, though most differences in AUC were within 95% confidence intervals. For children with Confirmed TB, model performance was best in India (AUC = 0.84; Fig. 4A–E) and worst in South Africa (AUC = 0.56). The model yielded AUCs of 0.71, 0.69, and 0.66 in children with Confirmed TB in Uganda, Peru, and The Gambia, respectively (Fig. 4D–E). Similar to the overall analysis, model performance was lower for the identification of Unconfirmed TB across countries, with AUCs ranging from 0.49–0.59.

### Machine Learning Model

**Figure 5:**
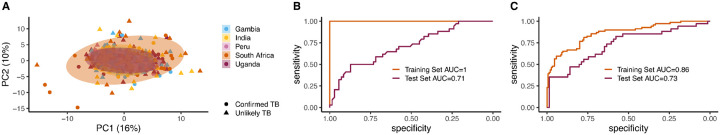
Metabolic signature performance using machine learning – (A) Principal component analysis showing the clustering of each country after Limma batch correction using the 105 metabolites detected and quantified in all cohorts. For the 7-metabolite signature selected by random forest, area under the receiver operator characteristic curve (AUC) was calculated using (B) random forest for model training and validation and (C) using support vector machine.

To see if model performance could be improved with different model building strategies, we implemented an alternative workflow using machine learning techniques. We first used Limma to correct for country-level and batch effects (Fig. 5A). We then used randomforestSRC for metabolite selection, which produced a list of seven metabolites: creatine, retinol, tryptophan, urate, dihyroxybenzoate, cortisol, and hydroxyphenylpropionate. Using these seven metabolites, we created machine learning models from the training data with random forest and SVM. The random forest and SVM models produced similar classification accuracy compared to the logistic regression model (AUC = 0.71 and 0.73 respectively; Fig. 5B and 5C).

### Metabolite concentrations and nutritional status

Given many of the metabolites in the 9-metabolite plasma signature are associated with dietary intake and nutritional status, we sought to determine whether any were associated with stunted growth or underweight status, independent of their association with TB disease. Only creatine was significantly associated with stunting (q = 0.03; Table 2) while creatine and tryptophan were both associated with being underweight (q = 0.17 and 0.15 respectively; Table 3). Metabolites significantly associated with both stunting and underweight status included creatinine, lauric acid, cystathionine, and β-hydroxybutyrate. Glutamate and the long chain fatty acids myristic acid, gondoic acid, and heptadecanoic acid were only associated with stunting while the metabolites arginine, inosine, tyrosine, hippurate, and arachidic acid were associated with being underweight. The 9-metabolite model had similar performance in children with and without stunting (Figure S3A) as well as children who were or were not underweight (Figure S3B).

## Discussion

In this study, we found that plasma metabolic signatures may have utility as biomarkers of TB disease in children. A plasma signature of tryptophan, creatine, alanine, fumarate, citrulline, retinol, cortisol, butyrylcarnitine, and nicotinamide was able to classify children with Confirmed TB with moderate accuracy across countries. This is, to our knowledge, the largest and most diverse study of children evaluated for TB disease for whom plasma metabolomics has been used to identify potentially diagnostic biomarkers. Previous studies have examined plasma metabolic signatures in children from a single center and have primarily focused on children greater than five years of age^16,17^. Here we examined metabolic biomarkers of TB in over 100 children with Confirmed TB with symptomatic controls across all age ranges from multiple geographic locations. This is critical, as we provide evidence that optimal blood-based molecular signatures of TB disease in children may differ by geographic location.

Diagnosing TB disease remains a clinical challenge, with an estimated 40% of cases never reported to public health programs^1^. Creation of point-of-care assays that can be used to diagnose TB using non-sputum biospecimens (i.e. blood or urine) without a laboratory is critical to closing this diagnostic gap^31^. The need is even greater for children, who are often unable to produce sputum for microbiologic testing and for whom current microbiologic tests are less sensitive than for adults^32^. While the plasma metabolic signatures identified in the present study did not meet the WHO target product profile (TPP) for a point-of-care TB diagnostic or TB triage test^7^, they did show moderate classification accuracy for children with Confirmed TB. Thus, it remains possible that these metabolites could be combined with other biomarkers to reach a classification accuracy in line with published TPPs. It is also important to consider these results in the context of currently available tools to diagnose TB disease. The performance of the metabolic signatures was similar to that of WHO treatment decision algorithms, which have also not met TPP thresholds in programmatic settings^33^.

This classification accuracy is substantially lower than in adults, where metabolic signatures have been much more accurate and consistent across populations^12,13,15,34,35^. The reasons for this difference are not clear but signature accuracy may have been impacted by greater sample collection protocol variation in this case-control study. We found strong site-specific effects in our pediatric cohorts, which overlapped with the anticoagulant type used for plasma collection. Across the five study sites included, three different anticoagulants were used for blood collection. This is important as prior studies have indicated that the type of tube used for blood collection is the single largest source for experimental variation in metabolomics studies^36^. However, we also found that even in countries where the same type of blood tube was used (e.g. India and The Gambia) there remained strong site-specific effects. While other sources of variation in study protocols can’t be ruled out, it does suggest site-specific metabolome variability had at least some impact on the classification accuracy.

There are also physiologic reasons why metabolic signatures could be less accurate in children versus adults. Prior work by our group and others has also shown that metabolic signatures in adults are associated with *Mtb* burden and disease severity^12,15,37^. Thus, it would not be unexpected that burden-dependent signatures are less accurate in children, who generally have paucibacillary disease. Data were corrected for age greater than or less than five years, but changes in metabolism associated with growth and diet may be more nuanced. International studies indicate plasma amino acid concentrations are more variable among even healthy children, particularly in the very young^38^. This has the potential to cause greater interindividual variation, thereby affecting the classification accuracy of diagnostic signatures relative to adults with TB disease. While it would be preferred that any signature for TB disease in children have equal performance across different populations^31^, host biomarker signatures can be adjusted to account for population-specific effects that maintain performance in different settings^39^. A similar strategy has been shown to improve accuracy of automated chest x-ray algorithms for TB triage^40^.

The signature in the present study contains multiple amino acids that have also been shown to differentiate children and adults with TB disease in independent studies.^12–14,34,41^ Decreased tryptophan is likely the result of increased indoleamine-2,3-dioxygenase expression, which plays an important immunomodulatory role in TB disease by breaking down tryptophan to kynurenine^42–44^. We found a plasma signature that included the kynurenine/tryptophan ratio plus retinol had similar classification accuracy to the kynurenine/tryptophan ratio alone in a prior pediatric TB study in India^16^. The modest performance of the kynurenine/tryptophan ratio as a biomarker of TB disease in children is in stark contrast to adults, where it has regularly been found to have excellent classification accuracy (AUC > 0.88).^12–15,34,35^ The role of the other amino acids in TB pathophysiology is less clear. One possibility we considered was that these changes were driven by malnutrition, which is highly co-prevalent with TB disease in children.^45^ Yet of the metabolites included in the final signatures, we found only creatine had a significant association with both stunting and underweight status, and TB disease classification accuracy did not differ in stunted and underweight children. Further, differences in plasma concentrations observed in different geographic locations were not accounted for by differences in nutritional status between populations. This suggest the metabolic responses observed were due to TB itself rather than the malnutrition that often accompanies the disease.

This study is subject to several limitations. Because the plasma samples analyzed were collected in different anticoagulants, only quantified metabolites could be compared across all five cohorts and strong batch effects remained. In future studies, it will be important to compare plasma metabolic signatures in children evaluated for TB disease using a universal collection protocol to minimize any variability introduced by the collection process. While most children diagnosed with TB disease were not microbiologically confirmed and thus may not have had TB disease, we did have over 100 participants that did have a confirmed TB diagnosis. However, even this relatively large sample size allows for limited subgroup analyses of conditions that are often co-prevalent in children with TB disease such as malnutrition and HIV.

In conclusion, we discovered plasma metabolic signatures distinguished children with Confirmed TB disease from those with Unlikely TB in diverse geographic areas. These signatures consisted of decreased concentrations of the amino acids tryptophan, citrulline, histidine, arginine, and alanine, as well as retinol and increased concentrations of cortisol, nicotinamide, and butyrylcarnitine. Yet the performance accuracy of these signatures did not meet the WHO TPP for a TB triage test and showed limited ability to distinguish children with Unconfirmed TB from those with Unlikely TB. Future prospective studies with a uniform collection protocol are needed to determine whether the classification accuracy of these signatures could be improved either alone or in combination with other biomarkers.

## Supplementary Material

This is a list of supplementary files associated with this preprint. Click to download.

- ComboAllCountriesQuantifiedMerged11082024signif.txt
- COMBOAllSitesClinicalData08022024.txt
- FigureS1.pdf
- FigureS2.pdf
- FigureS3.pdf

## Figures and Tables

**Tables 1 –:** Participant clinical and demographic characteristics

	All	Peru	Uganda	India	Gambia	South Africa
	(n=438)	(n=71)	(n=107)	(n=109)	(n=99)	(n=52)
TB classification, n(%)						
Confirmed TB	104 (24)	17 (24)	17 (16)	19 (17)	29 (29)	22 (42)
Unconfirmed TB	108 (25)	19 (27)	32 (30)	29 (27)	19 (19)	9 (17)
Unlikely TB	226 (51)	35 (49)	58 (54)	61 (56)	51 (52)	21 (40)
Age categories, n (%)						
< 5 years	229 (52)	31 (44)	64 (60)	71 (65)	71 (72)	47 (90)
^3^ 5 years	209 (48)	40 (56)	43 (40)	38 (35)	28 (28)	5 (10)
Female sex, n (%)	202 (46)	34 (48)	49 (46)	48 (44)	51 (52)	20 (38)
HIV positive, n (%)	28 (6)	0 (0)	7 (7)	1 (1)	0 (0)	20 (38)
Weight for age z-score^a^, median (IQR)	−1.0 (−1.8, −0.2)	0.0 (−0.6, 0.5)	−0.6 (−1.2, −0.1)	−1.4 (−2, −0.9)	−1.4 (−1.8, −0.5)	−1.4 (−2.6, −0.2)
Height for age z-score^b^, median (IQR)	−0.9 (−1.7, −0.06)	−0.6 (−1.2, −0.1)	−1.1 (−2.0, −0.2)	−0.6 (−1.5, 0.0)	−0.6 (−1.7, 0.0)	−1.9 (−2.4, −0.9)
Underweight, n (%)	72 (16)	0 (0)	17 (16)	25 (23)	13 (13)	17 (33)
Stunted, n (%)	80 (18)	3 (4)	27 (25)	15 (14)	11 (11)	24 (46)

a Includes 358 participants with available data

b Includes 424 participants with available data

**Table 2 –:** Metabolites associated with stunting

Metabolite	Non-Stunted Mean, mM	Stunted Mean, mM	p-value	adjusted p-value
**creatinine**	26.8	20.5	0.0003	0.027
**lauric acid**	6.2	12.6	0.0006	0.027
**creatine**	63.4	86.6	0.0008	0.027
**cystathionine**	0.054	0.068	0.0069	0.180
**b-hydroxybutyrate**	236	464	0.0125	0.188
**myristic acid**	9.4	15.6	0.0127	0.188
**glutamic acid**	116	124	0.0131	0.188
**gondoic acid**	10.8	16.1	0.0150	0.188
**heptadecanoic acid**	1.6	2.3	0.0161	0.188

a Stunted = HAZ < −2.0, Non-stunted = HAZ ^3^ −2.0

b Includes 424 participants with available data

**Table 3 –:** Metabolites associated with underweight status

Metabolite	Not Underweight Mean, mM	Underweight Mean, mM	p-value	adjusted p-value
**cystathionine**	0.048	0.091	1.33E-05	0.001
**hydroxyphenylpropionate**	0.17	0.26	0.001	0.076
**lauric_acid**	7.0	13.8	0.003	0.119
**b-hydroxybutyrate**	259	491	0.004	0.119
**arginine**	54.3	46.4	0.009	0.154
**tryptophan**	47.3	40.4	0.009	0.154
**inosine**	2.8	4.2	0.013	0.169
**creatine**	70.5	85.3	0.013	0.169
**creatinine**	23.7	21.2	0.016	0.169
**tyrosine**	67.1	61.9	0.018	0.169
**hippurate**	3.9	4.3	0.019	0.169
**arachidic_acid**	27.0	31.5	0.019	0.169

a Underweight= WAZ < −2.0, Not Underweight = WAZ ^3^ −2.0

b Includes 358 participants with available data

## Data Availability

Data is provided within the manuscript or supplementary information files
